# Orofacial Pains

**DOI:** 10.1155/2017/1915691

**Published:** 2017-11-16

**Authors:** Vahid Rakhshan, Francesca A. Bianchi, Lucindo J. Quintans-Júnior, Aurelio A. Alonso

**Affiliations:** ^1^Department of Cognitive Neuroscience, Institute for Cognitive Science Studies, Tehran, Iran; ^2^Department of Dental Anatomy, Faculty of Dentistry, Islamic Azad University, Tehran, Iran; ^3^Programme in Experimental Medicine and Therapy, Doctoral School in Life and Health Sciences, Maxillofacial Surgery Section, Surgical Sciences Department, S. Giovanni Battista Hospital, University of Turin, Turin, Italy; ^4^Laboratory of Neuroscience and Pharmacological Assays, Federal University of Sergipe, São Cristóvão, SE, Brazil; ^5^Orofacial Pain Division, Duke Center for Translational Pain Medicine, Department of Anesthesiology, School of Medicine, Duke University, Durham, NC, USA

Orofacial pains refer to pains experienced in the mouth, face, and head regions [1, 2]; they might stem from disorders and diseases of the temporomandibular joint and masticatory musculature, head and neck muscle tension, neurovascular disorders (such as migraine), neuropathic disorders (such as trigeminal neuralgia), inflammations in the area (such as sinusitis or dental pulpitis), or dentomaxillofacial treatments [1–7]. They represent a serious clinical problem: they have a broad range of origins and characteristics, many of them have noticeable intensities (e.g., comparable to spinal pain disorders), and they are very common (as out of every five persons, one might experience them) [3, 8]. Some of these pains can be quite debilitating, impairing emotional, social, and physical functioning, reducing satisfaction with life, and, in certain cases, discouraging patients from seeking or continuing treatment [1, 3, 4, 7, 9]. Multifactorial and complex mechanisms underlying many orofacial pains and risk factors associated with them are not completely understood [3, 5, 8, 10]. The identification of their etiologies and risk factors and development of more effective therapeutic modalities and medications to prevent, control, or eliminate them are of significant value and hence targets for ever-increasing research [3–6, 8, 10–12]. This special issue sought to review and publish quality articles on nature and remedies of a group of orofacial pains which might be more relevant to the field of dentistry, such as temporomandibular disorders or iatrogenic pains.

In this issue, readers will find nine articles comprising “Trigeminal Neuralgia, Glossopharyngeal Neuralgia, and Myofascial Pain Dysfunction Syndrome: An Update” by M. Khan et al. (the authors summarized the currently available diagnostic procedures and treatment options for trigeminal neuralgia, glossopharyngeal neuralgia, and myofascial pain dysfunction syndrome); “The Evaluation of the Clinical Effects of Botulinum Toxin on Nocturnal Bruxism” by F. Asutay et al. (a clinical study on the role of botulinum toxin in the management of bruxism); “A Preliminary Genome-Wide Association Study of Pain-Related Fear: Implications for Orofacial Pain” by C. L. Randall et al. (a GWAS research on genetic factors [as well as other risk factors like age, sex, and education] contributing to fears of medical/dental pain, severe pain, and minor pain); “Sensitivity, Specificity, Predictive Values, and Accuracy of Three Diagnostic Tests to Predict Inferior Alveolar Nerve Blockade Failure in Symptomatic Irreversible Pulpitis” by D. Chavarría-Bolaños et al. (a research about diagnostic values of tests used to predict the failure of inferior alveolar nerve block); “Immediate Postoperative Pain and Recovery Time after Pulpotomy Performed under General Anaesthesia in Young Children” by S. Keles and O. Kocaturk (this study compared postoperative pain scores after pulpotomy with pain after filling-only treatments performed under general anaesthesia‎); “Prevalence of Painful Temporomandibular Disorders and Correlation to Lifestyle Factors among Adolescents in Norway” by V. Østensjø et al. (a research on the prevalence and risk factors of painful temporomandibular disorders among adolescents); “Temporomandibular Disorders and Headache: A Retrospective Analysis of 1198 Patients” by C. Di Paolo et al. (an article on the positive association between headaches such as migraine or tension headache with symptoms of temporomandibular disorders); “Central Sensitization-Based Classification for Temporomandibular Disorders: A Pathogenetic Hypothesis” by A. Monaco et al. (the authors hypothesized an alternative classification for temporomandibular disorders based on the presence of central sensitization and on individual response to sensory ultralow frequency transcutaneous electrical nerve stimulation); and finally the article “Effectiveness of Low-Level Laser Therapy in Reducing Orthodontic Pain: A Systematic Review and Meta-Analysis” by N. F. Deana et al. (a meta-analysis of the effect of near infrared low-level lasers on orthodontic pain).

*Vahid Rakhshan* *Francesca A. Bianchi* *Lucindo J. Quintans-Júnior* *Aurelio A. Alonso*
